# Physically Crosslinked Chondroitin Sulfate (CS)–Metal Ion (M: Fe(III), Gd(III), Zn(II), and Cu(II)) Particles for Versatile Applications and Their Biosafety

**DOI:** 10.3390/ph16040483

**Published:** 2023-03-23

**Authors:** Selin S. Suner, Mehtap Sahiner, Evrim Umut, Ramesh S. Ayyala, Nurettin Sahiner

**Affiliations:** 1 Department of Chemistry, Nanoscience and Technology Research and Application Center, Canakkale Onsekiz Mart University, Terzioglu Campus, 17100 Canakkale, Turkey; 2 Department of Bioengineering, Engineering Faculty, Canakkale Onsekiz Mart University, 17100 Canakkale, Turkey; 3 Department of Medical Imaging Techniques, School of Healthcare, Dokuz Eylul University, 35330 Izmir, Turkey; 4 Bioİzmir-Izmir Health Technologies Development and Accelerator Research and Application Center, Dokuz Eylul University, 35330 Izmir, Turkey; 5 Department of Ophthalmology, Morsani College of Medicine, University of South Florida, 12901 Bruce B Downs B. Downs Blv, MDC 21, Tampa, FL 33612, USA; 6 Department of Chemical and Biomolecular Engineering, University of South Florida, Tampa, FL 33620, USA

**Keywords:** chondroitin sulfate (CS), CS–metal ion particles, biocompatible, antibacterial, magnetic resonance imaging (MRI)

## Abstract

Chondroitin sulfate (CS), a well-known glycosaminoglycan, was physically crosslinked with Fe(III), Gd(III), Zn(II), and Cu(II) ions to obtain CS-Fe(III), CS-Gd(III), CS-Zn(II), and CS-Cu(II) polymeric particles for multipurpose biological applications. The CS–metal ion-containing particles in the micrometer to a few hundred nanometer size range are injectable materials for intravenous administration. The CS–metal ion-containing particles are safe biomaterials for biological applications because of their perfect blood compatibility and no significant cytotoxicity on L929 fibroblast cells up to a 10 mg/mL concentration. Furthermore, CS-Zn(II) and CS-Cu(II) particles show excellent antibacterial susceptibility, with 2.5–5.0 mg/mL minimum inhibition concentration (MIC) values against *Escherichia coli* and *Staphylococcus aureus*. Moreover, the in vitro contrast enhancement abilities of aqueous CS–metal ion particle suspensions in magnetic resonance imaging (MRI) were determined by obtaining T_1_- and T_2_-weighted MR images using a 0.5 Tesla MRI scanner and by calculating the water proton relaxivities. Therefore, these CS-Fe(III), CS-Gd(III), CS-Zn(II), and CS-Cu(II) particles have significant potential as antibacterial additive materials and MRI contrast enhancement agents with less toxicity.

## 1. Introduction

As a sulfated glycosaminoglycan, chondroitin sulfate (CS) has multiple functions in the body, including structural and signaling functions [1,2]. It usually covalently links to proteins [3]. CS is extensively consumed orally by humans and non-humans as it is believed to be favorable for those with joint-related diseases [4]. Capsules and tablets of CS are taken orally, and it is used as an additive in foods and beverages, creams, eye drops, cosmetics, and medical applications [4]. Furthermore, CSs are widely used in other pharmacology-based applications, including coatings for implants, hydrogels for controlled-release drug delivery, scaffolds for tissue engineering, and the diagnosis of certain diseases [5,6,7,8]. It has been stated that dopamine-functionalized CS hydrogels are suitable for the reconstruction of cartilage tissues with their mucoadhesive properties [9]. There are also studies on the use of CS-based fibers in tissue engineering [10,11]. CS has been used to coat magnetite nanoparticles to prepare them in the fabrication of biocompatible magnetic fluid [12]. Polyelectrolyte CS microgels have also been prepared as a carrier of antioxidant material [13].

Glycosaminoglycans as CS is generally used in biomedical applications due to their non-toxic, biocompatible, biodegradable nature in addition to highly beneficial biological functions in wound healing, infection, growth factor signaling, cell growth, and osteoarthritis [14]. This biomolecule has the ability to make complexes with different metal ions that may render additional biological advantages in different areas. CS derivatives have been modified with several metal ions to enhance their efficiency against osteoarthritis and osteoporosis, such as calcium, strontium, and magnesium. The CS–Ca complex exhibited strong antiosteoporosis properties [15]. SrCS metal complexes are safe for chondrocytes and osteoblasts, and can increase collagen production and reduce inflammation [16]. In order to regenerate bone, chitosan–strontium–CS has been developed [17]. In another study, ion exchange was used to prepare the chondroitin sulfate–zinc (CSZn) complex. It is used as a wound-healing material with antibacterial and anti-inflammatory properties [18]. Bioactive magnesium phosphate–CS composites are designed as inorganic bone fillers [19]. MgCS can increase osteoarthritis chondrocyte proliferation and decrease apoptosis [20]. A doxorubicin hydrochloride carrier was generated by coating superparamagnetic iron oxide nanoparticles with CS [19]. Apart from these, it has been stated that CS chelates with Cu ions [21].

Although there are some studies in the literature proposing CS–magnetic ion or CS–magnetic nanoparticle composites for use as contrast agents in magnetic resonance imaging (MRI) [12], none of them evaluate MRI contrast enhancement performance. Celli et al. showed the synthesis of CS-Fe(II) and/or CS-Fe(III) complexes [22]. In another study, Werner et al. reported the preparation of CS-Gd(III) complexation as a contrast agent for MRI-based relaxometry [23]. Similarly, a CS-Cu(II) complex was prepared and characterized in a study [24]. In addition, a CS-Zn(II) complex solution was designed for wound healing applications with antibacterial properties [18]. These studies supported that cationic Fe(III), Gd(III), Zn(II), and Cu(II) ions could interact with anionic CS polymers to afford CS–metal ion complexes. It is well known that the complexation between the carbohydrate and metal ions is expressed as physically crosslinked materials [25,26,27]. However, in this study, we report the synthesis of physically crosslinked CS-Fe(III), CS-Gd(III), CS-Zn(II), and CS-Cu(II) particles in a microemulsion medium, show their potential use in diverse applications such as antibacterial activity, and thoroughly investigate their MRI contrast enhancement capabilities. The CS–metal ion particles were characterized in terms of morphology, size distribution, zeta potential, chemical structure, and thermal degradation. Furthermore, the CS–metal ion particles were tested for blood compatibility via hemolysis and blood clotting assays. In addition, the cytotoxicity of the CS–metal ion particles was also determined on healthy L929 fibroblast cells. The minimum inhibition concentration (MIC) values of the CS–metal ion particles were determined against Gram-positive and Gram-negative bacteria strains by using a microtiter assay to assess the antibacterial susceptibility of the particles. In order to test the in vitro MRI contrast enhancement capabilities of the CS–metal ion particles, a series of T_1_- and T_2_-weighted MR images were obtained on phantoms including the particles’ aqueous suspensions, and by analyzing the images, the water proton relaxivities were calculated.

## 2. Results and Discussion

**Figure 1:**
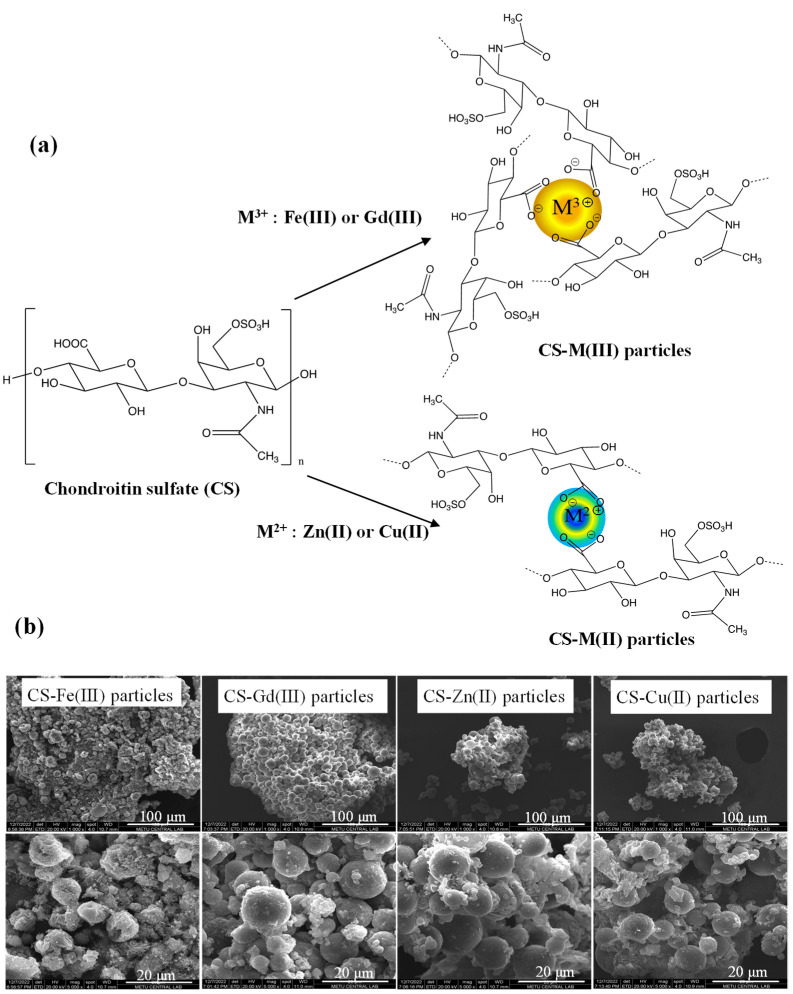
(**a**) Schematic representation of synthesis reaction of CS-M(III) and CS-M(II) particles and (**b**) their SEM images.

Chondroitin sulfate (CS) is a well-known sulfate group containing glycosaminoglycan. As seen in Figure 1a, deprotonated carboxylic acid groups of linear CS polymer in 0.1 M NaOH solution can ionically interact with metal ions in 0.1 M HCl solutions of three valent metal ions such as Fe(III) and Gd(III) ions or two valent metal ions such as Zn(II) and Cu(II) ions as crosslinkers. To prepare spherical CS–metal(III) or CS–metal(II) particles, the physical crosslinking reaction was accomplished in a reverse micelle microemulsion system. These studies supported that cationic Fe(III), Gd(III), Zn(II), and Cu(II) ions could interact with anionic CS polymers to afford CS–metal ion complexes. In the synthesis of CS-Gd(III), CS-Fe(III), CS-Zn(II), and CS-Cu(II) microgels, 1.74 μg of Gd(III), 1.53 μg of Fe(III), 1.28 μg of Zn(II), and 0.98 μg of Cu(II) ions were used that are stoichiometrically the same mol ratio relative to the repeating units of the used CS: 1 mg linear CS. In this study, the crosslinking reaction, which is a complexation between the carboxylic acid groups of the CS polymer and these metal ions, was performed in microemulsion systems to attain spherical CS–metal ion polymeric particles to obtain particles of an injectable size range with two different biological uses, e.g., as an MRI contrast agent and as antibacterial additive materials.

The size and morphology of the CS-Fe(III), CS-Gd(III), CS-Zn(II), and CS-Cu(II) particles were assessed via SEM images, as shown in Figure 1b. The SEM images of the CS-based particles revealed that the CS-Fe(III), CS-Gd(III), CS-Zn(II), and CS-Cu(II) particles have almost spherical shapes of 0.5–20 μm in diameter.

**Figure 2:**
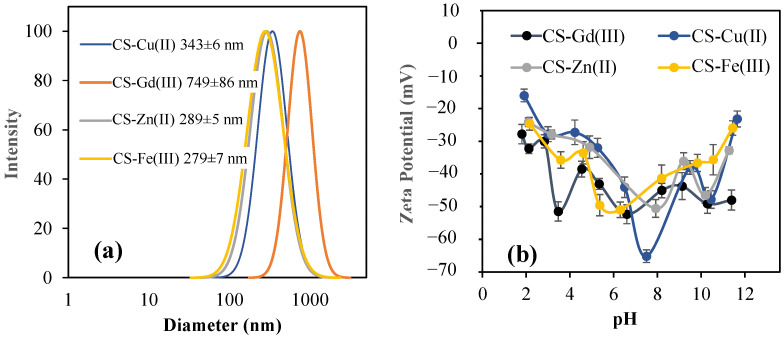
(**a**) Size distribution of CS-Fe(III), CS-Gd(III), CS-Zn(II), and CS-Cu(II) particles using DLS measurements, and (**b**) their zeta potential values at pH range from 2 to 10.

The DLS size analysis of the CS–metal ion particles filtered with 5 µm filter paper is given in Figure 2a. The average size distribution of the CS-Fe(III), CS-Gd(III), CS-Zn(II), and CS-Cu(II) particles was measured as 279 ± 7 nm, 794 ± 86 nm, 289 ± 5 nm, and 343 ± 6 nm, respectively. The zeta potential values for the CS-Fe(III), CS-Gd(III), CS-Zn(II), and CS-Cu(II) particles were measured in pH 2–12 solutions, as shown in Figure 2a. The pH of the aqueous solutions of the CS-Fe(III), CS-Gd(III), CS-Zn(II), and CS-Cu(II) particles was measured as 6.3, 8.2, 7.9, and 7.5, respectively. Their zeta potential values were also measured as −50.9 ± 2.3, −45.0 ± 2.2, −50.6 ± 2.7, and −65.2 ± 1.9 mV, suggesting the high stability of the particles in aqueous environments. Because the sulfate functional groups are highly ionizable in aquatic environments, the high zeta potential values for CS–metal ion particles are reasonable.

**Figure 3:**
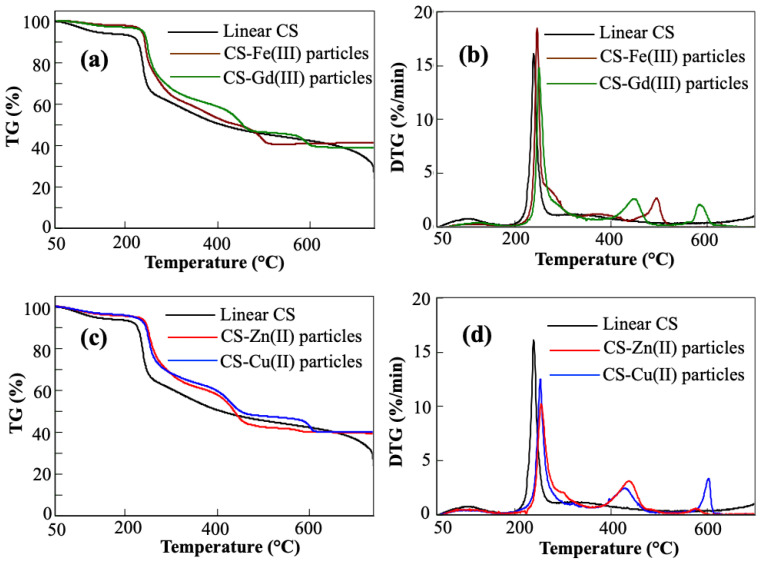
Thermal degradation% (TG%) and differential thermal degradation (DTG) %/min of (**a**,**b**) linear CS, CS-Fe(III), CS-Gd(III), and (**c**,**d**) CS-Zn(II) and CS-Cu(II) particles.

Thermogravimetric (TG) analysis and differential thermogravimetric (DTG) analysis of linear CS and the CS–metal ion particles were also carried out, as shown in Figure 3. It was determined that all types of particles were slightly degraded at about 100 °C because of the evaporation of the bound water, and one main degradation in the range of 215–260 °C with almost 34.5, 23.6, 21.3, 18.7, and 22.3% weight loss values for the linear CS, CS-Fe(III), CS-Gd(III), CS-Zn(II), and CS-Cu(II) particles, respectively. This maximum peak at about 240 °C corresponds to the decomposition of CS. Among these degradations, the linear CS (non-crosslinked CS) shows two degradations in the temperature ranges of 260–420 °C with 50.8%wt loss and at 650–700 °C with 63.5% wt loss. In the complex forms, the CS-Fe(III) particles had two more degradations with maximum peaks at 371 °C with 43.6% wt loss and 495 °C with 56.5.5% weight loss. Similarly, the CS-Gd(III) particles revealed two more slight degradations at 453 °C with 49.7% wt loss and at 586 °C with 57.6% wt loss.

As shown in Figure 3c,d, the CS-Zn(II) and CS-Cu(II) particles show almost similar degradation curves at 255, 437, and 603 °C maximum decomposition peaks with 14.9, 49.9, and 59.4% wt loss values for the CS-Zn(II) particles and 18.8, 47.6, and 58.2% weight loss values for the CS-Cu(II) particles, respectively. At 700 °C, the remaining weight % of linear CS of 36.5% was measured as 41.2, 38.9, 39.5, and 41.1% for the CS-Fe(III), and CS-Gd(III), CS-Zn(II), and CS-Cu(II) particles, respectively. The difference between the CS and CS–metal ion particles represents a metal ion content of 4.7 wt% Fe(III), 2.4 wt% Gd(III), 3.0 wt% Zn(II), and 4.6 wt% Cu(II) ions.

**Figure 4:**
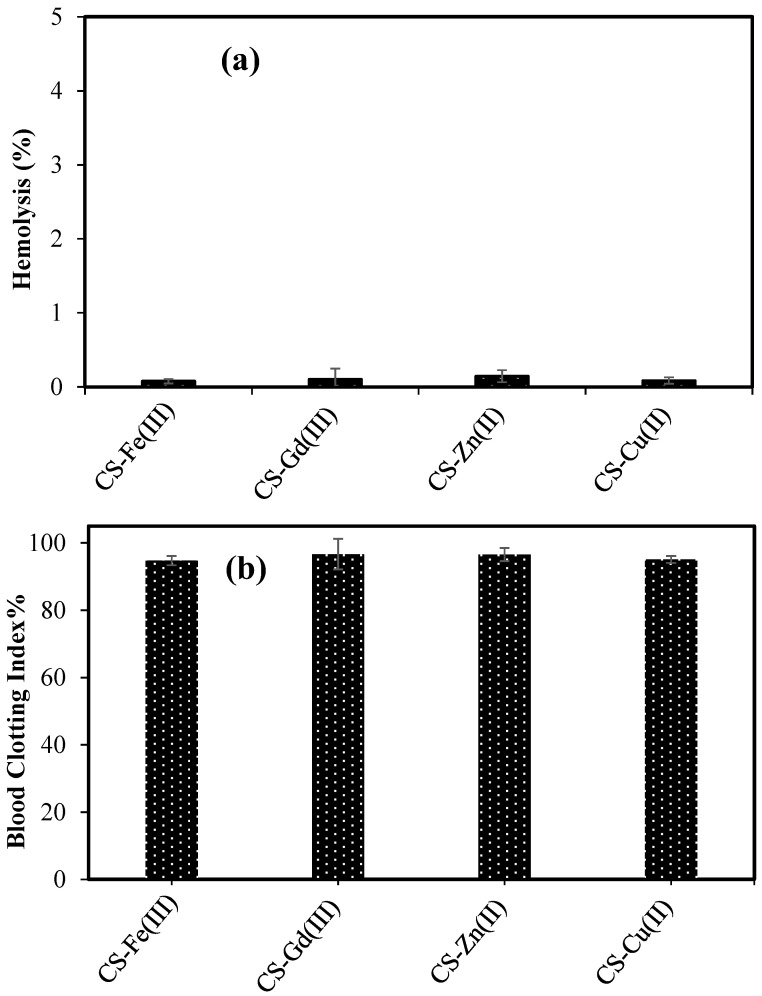
(**a**) Hemolysis ratio and (**b**) blood clotting index of CS-Fe(III), CS-Gd(III), CS-Zn(II), and CS-Cu(II) particles at 1 mg/mL concentration.

The hemocompatibility of the prepared CS–metal(III) and CS–metal(II) particles was investigated through hemolysis and blood clotting tests. As presented in Figure 4a, the hemolysis ratio of the CS-Fe(III), CS-Gd(III), CS-Zn(II), and CS-Cu(II) particles at a 1 mg/mL concentration was found to be 0.07 ± 0.02, 0.10 ± 0.14, 0.14 ± 0.08, and 0.08 ± 0.04%, respectively. According to the literature, a hemolysis ratio of up to 2% indicates non-hemolytic materials [28], and these results signified that all types of CS–metal ion-based particles are non-hemolytic up to a concentration of 1 mg/mL.

The effect of the materials on the blood clotting mechanism is another important parameter of blood compatibility. As illustrated in Figure 4b, the blood clotting index values of the CS-Fe(III), CS-Gd(III), CS-Zn(II), and CS-Cu(II) particles at a 1 mg/mL concentration were measured as 94.7 ± 1.34, 96.6 ± 4.5, 96.5 ± 1.9, and 95.1 ± 1.0, respectively. Biomaterials designed for intravenous applications should not interfere with the blood clotting mechanism of the body. Therefore, materials with high blood clothing values are assumed to be materials that do not interfere with the blood and are accepted as being blood compatible [29,30]. These results confirm that all types of CS–metal ion-based particles do not interfere with the clotting mechanisms up to a concentration of 1 mg/mL when in contact with the blood. It is obvious that CS-Fe(III), CS-Gd(III), CS-Zn(II), and CS-Cu(II) particles are safe materials for intravenous application because of their low hemolysis ratio and high blood clotting values. As reported by Ramalho et al., free Gd(III) ions show toxicity because they biologically compete with Ca(II) ions, which plays a significant role in many physiological processes, especially in the blood coagulation mechanisms. In addition, Gd(III) ions possess slow systemic excretion [31]. Another study revealed that a clinically used Gd contrast agent injection significantly inhibited white blood cells [32]. The prepared CS-Fe(III) and CS-Gd(III) may be used as MRI contrast enhancing agents instead of toxic paramagnetic bare metal ions such as Gd(III) or their chelating forms such as gadolinium–diethylene triamine penta-acetic acid [31,33].

**Figure 5:**
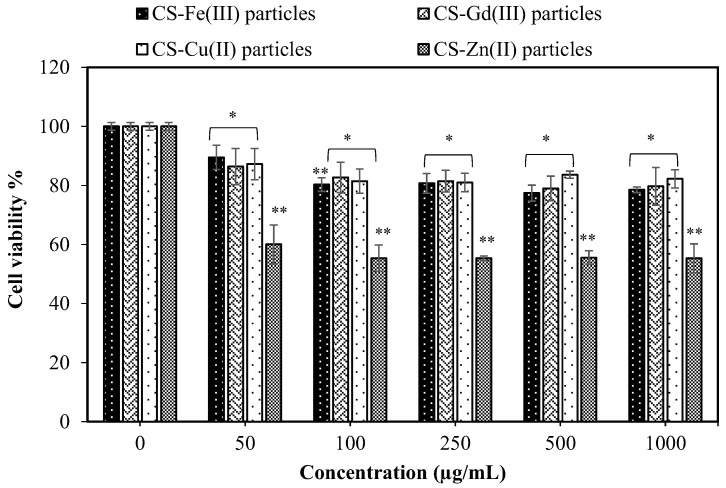
Cytotoxicity of CS-Fe(III), CS-Gd(III), CS-Zn(II), and CS-Cu(II) particles against L929 fibroblast cells at 24 h incubation time (Values are expressed as mean ± SD, n = 3, * *p*-value < 0.05, and ** *p*-value < 0.001 compared with control group).

The cytotoxicity of the materials is also very important for in vivo applications. Therefore, the most common analysis to observe the biocompatibility of the materials and the cell viability percentage of the CS–metal ion-based particles was examined on the L929 fibroblast cells at 24 h incubation time from 50 to 1000 μg/mL concentration range. As shown in Figure 5, the viability of the fibroblasts in the presence of CS-Fe(III), CS-Gd(III), CS-Zn(II), and CS-Cu(II) particles at a 50 μg/mL concentration was found to be 89.5 ± 4.1, 86.4 ± 6.1, 60.0 ± 6.5, and 87.2 ± 5.3%, respectively, and these cell viability values did not significantly change even at a particle concentration of 1000 μg/mL.

It is clear that all types of CS–metal-based particles except the CS-Zn(II) particles show no significant toxicity on the fibroblasts up to a 1000 μg/mL concentration and, therefore, they could be used as tissue-contacting materials with antibacterial properties. According to the synthesis of the microgels, 1 mg of CS-Gd(III) microgels can contain a maximum of 1.53 mg of Gd(III) ions. In the clinical use of Gd-DO3A-butrol (Gadovist^®^, Bayer Healthcare), the recommended dose is 0.1 mL/kg body weight at 1 mmol/mL concentration of gadobutrol containing 157.2 mg Gd(III) ions [34], which is almost ten-fold higher than Gd(III)s ion in our CS-Gd(III) microgels. These results indicate that CS-Gd(III) microgels have great potential as a contrast enhancing agent in MRI applications with their low toxicity in the blood and fibroblast cells up to a 1 mg/mL concentration.

The antibacterial effects of CS-Fe(III), CS-Gd(III), CS-Zn(II), and CS-Cu(II) particles against Gram-negative *Escherichia coli* ATCC 8739 and Gram-positive *Staphylococcus aureus* ATCC 6538 for a 24 h incubation were examined using a microtiter test, and their minimum inhibition concentration (MIC) values are listed in Table 1.

The MIC values of the CS-Zn(II) and CS-Cu(II) particles were determined as 2.5 and 5.0 mg/mL, respectively, against *E. coli*, but slightly higher MIC values of 5.0 mg/mL were obtained against *S. aureus* for both materials. Furthermore, the CS-Fe(III) particles showed a 5.0 mg/mL MIC value against *E. coli*, but no antibacterial effects against *S. aureus.* Among these materials, the CS-Gd(III) particles did not provide any antibacterial activity for either bacterial species. Some metal ions including Fe(III), Zn(II), and Cu(II) ions afford perfect antibacterial activity on a wide range of microorganisms due to their toxic effect on bacteria, fungi, and viruses [35]. According to our results, the MIC values against *E. coli* for the Fe(III), Zn(II), and Cu(II) ions were determined as 0.5, 0.1, and 0.1 mg/mL, respectively. Furthermore, the MIC values of the Zn(II) and Cu(II) ions against *S. aureus* were determined as 0.1 and 0.3 mg/mL, respectively. However, their toxicity on other cells including healthy cells is limited, so they can be used as an antibacterial agent [36]; generally, the use of composite forms with biomacromolecules [37] or nanoparticle forms reduces the toxicity and enables a high antibacterial effect [38,39]. The prepared CS-Zn(II) and CS-Cu(II) particles exhibited excellent antibacterial ability on both Gram-positive and Gram-negative bacteria, with less or no toxicity to healthy cells. These results indicate that the CS-Zn(II) and CS-Cu(II) particles could be used as additive materials for antibacterial applications.

**Figure 6:**
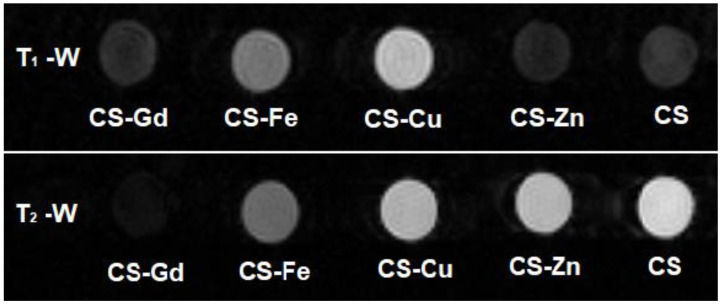
T_1_-weighted (**top**) and T_2_-weighted (**bottom**) MR images of CS–metal and CS microgel water dispersions obtained with 0.5 T MRI scanner. (T_1_-weighted image IR sequence parameters: TR = 5000 ms, TE = 20 ms, TI = 1000 ms; T_2_-weighted image SE sequence parameters: TR = 5000 ms, TE = 26 ms).

The MRI contrast enhancement efficiencies of the CS-based samples were investigated by obtaining MR images on water suspensions of CS-Gd(III), CS-Fe(III), CS-Cu(II), CS-Zn(II), and CS microgels with a 0.5 T MRI scanner. An MRI contrast depends on the number of hydrogen nuclei (protons) per unit volume as well as longitudinal and transverse relaxation times—the T_1_ and T_2_ of protons. Figure 6 shows T_1_-weighted and T_2_-weighted images recorded with inversion recovery (IR) and spin echo (SE) sequences, respectively. Qualitatively speaking, in Figure 6, the microgels including paramagnetic ions, that is, CS-Gd(III), CS-Fe(III), and CS-Cu(II), show brighter (positive) contrast in the T_1_-weighted image and darker (negative) contrast in the T_2_-weighted image compared to the “non-magnetic” gels, namely, CS-Zn(II) and CS. This is somewhat expected, because microgels including paramagnetic ions shorten the T_1_ and T_2_ values of nearby water protons in the suspension, which gives rise to enhanced contrast in MRI. Here, one should pay attention to the fact that the predominant contribution to the MR signal comes from bulk water protons, although there are water protons entrapped in swollen microgels and the protons of the CS matrix. Such a contrast enhancement effect is quantified with proton relaxivity r_1,2_, which is defined as the increment of the proton relaxation rate 1/T_1,2_ per 1 mM concentration of the magnetic ions (Equation (1)):

(1) $$r_{1,2}=\frac{1}{C_{\mathrm{param}}}\left(\frac{1}{T_{1,2\mathrm{param}}}-\frac{1}{T_{1,2\mathrm{pure}}}\right)$$

As can be seen in Equation (1), the calculation of proton relaxivities, as a measure of the contrast enhancement performance of the CS–metal microgels, requires individual T_1_ and T_2_ values, which can be deduced by analyzing a set of MR images (as in Figure 6) repeated for different sequence parameters. In particular, the signal intensity obtained with the spin echo (SE) sequence is given in Equation (2):

(2) $$S=\mathrm{PD}\left[1-\exp\left(-\frac{\mathrm{TR}}{T_{1}}\right)\right]\exp\left(-\frac{\mathrm{TE}}{T_{2}}\right)$$

In Equation (2), S is the signal intensity, PD is the proton density, and TR and TE are the repetition time and echo time, respectively. For this case, TR >> T_1_ Equation (2) can be written as S = PDexp(−TE/T_2_), and for a set of images repeated with progressively changing TE values, one can obtain T_2_ values for each gel simply by reading the signal intensities over a region-of-interest (ROI) selected on the corresponding gel (bright spot) on the image (see Supplementary Figure S1). Similarly, for the inversion recovery (IR) sequence, the signal intensity is given in Equation (3):

(3) $$S=\mathrm{PD}\left[1-2\exp\left(-\frac{\mathrm{TI}}{T_{1}}\right)+\exp\left(-\frac{\mathrm{TR}}{T_{1}}\right)\right]$$

In Equation (3), TI stands for the inversion time, and assuming TR>>T_1_, the expression converges to S = PD [1 − 2exp(−TI/T_1_)]; hence, T_1_ values for gels can be obtained by reading the signal intensities over the corresponding ROIs on images that were recorded with an IR sequence for different TI values.

**Figure 7:**
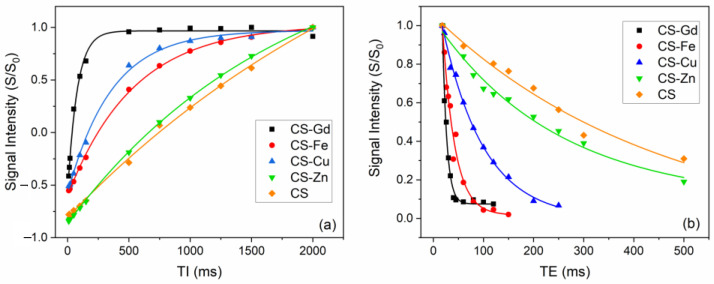
Time evolution of normalized signal intensities read on set of MRI images of CS–metal and CS microgel suspensions obtained with (**a**) inversion recovery (IR) and (**b**) spin echo (SE) sequences (see Supplementary Figure). Solid lines show mono exponential fits using Equations (2) and (3).

Figure 7a,b shows the normalized signal intensities as a function of TI and TE values for the IR and SE sequence, respectively (please note that in Figure 7a, the signal intensity starts from negative values due to the reversal of proton magnetization following the 180° excitation pulse in the IR sequence).

As a result of the theoretical fits shown in Figure 7 (according to Equations (2) and (3) for the case TR >> T1), Table 2 lists the water proton T_1_ and T_2_ values of the microgel samples, together with the proton relaxivities r_1,2_ calculated using Equation (1). One can clearly see that CS-Gd(III) has the highest longitudinal and transverse relaxivities among the others, likely due to the high effective magnetic moment of Gd approximately equal to 8 μ_B_ (Bohr magneton). In particular, its longitudinal relaxivity value r_1_ = 13.1 s^−1^·mM^−1^ is significantly higher than the commercial Gd-based contrast agents, i.e., r_1_ = 9.2 s^−1^·mM^−1^ for Gd-BOPTA (Multihance®, Bracco, NJ, USA), r_1_ = 6.1 s^−1^·mM^−1^ for Gd-DO3A-butrol (Gadovist®, Bayer Healthcare, Leverkusen, North Rhine-Westphalia, Germany), r_1_ = 4.3 s^−1^·mM^−1^ for Gd-DOTA (Dotarem^®^ Guerbet), and r_1_ = 3.8 s^−1^·mM^−1^ for Gd-DTPA (Magnevist^®^, Bayer HealthCare) at 0.5 Tesla [40]. However, this high longitudinal relaxivity does not reflect the positive contrast enhancement performance also due to the very short T_2_ of this sample (so-called T_2_-masking) (see Figure 6).

Surprisingly, despite Cu(II) having an effective magnetic moment μ_eff_ ≅ 2 μB that is smaller than Fe(III) μ_eff_ ≅ 6 μB, the CS-Cu(II) sample shows higher r1 contrast with respect to CS-Fe(III), consistent with Figure 6. On the other hand, the water proton transverse relaxivity for CS-Fe(III) is two times higher than CS-Cu(II). As expected, CS-Zn(II) has ignorable relaxivity values since Zn(II) does not have any unpaired electron; hence, its magnetic moment is zero.

For MRI studies, one can tentatively conclude that the magnetic CS–metal microgels act much like superparamagnetic nanoparticles (i.e., SPIO suspensions) by having an r_2_/r_1_ ratio higher than 2 (negative contrast agents) and showing r_2_ relaxivities roughly proportional to their magnetic moment. In such cases, the diffusion of water molecules under the stray field of the magnetized microgels’ so-called “outer sphere” relaxation mechanism is effective in the observed water proton relaxation [41].

For suspensions containing paramagnetic ions, the water proton relaxivity r_1_ depends on many aspects, such as: (i) the magnetic moment of the paramagnetic ions, (ii) the distance between a water molecule and a paramagnetic ion, (iii) the number of water molecules coordinated to a paramagnetic ion, (iv) the water residence time (or so-called chemical exchange time), and (v) the rotational correlation time of Gd-containing species [42]. In our opinion, compared with commercially available molecular Gd-based agents, our CS-Gd(III) particles have advantages, especially in terms of particle size and the number of Gd(III) ions per particle. The synthesized CS-Gd(III) particles have an approximate size of around 800 nm (see Figure 2), which means that, according to the Stokes–Einstein equation, they have a rotational correlation time much bigger than the one for molecular Gd-based agents that are in the size range of from few nanometers to tens of nanometers depending on the macrocyclic ligand chelating the Gd(III) ion. Hence, the slower rotation (i.e., slower time modulation of Gd–water proton dipolar coupling) causes higher r1 relaxivity. The second advantage of having a relatively larger particle size is that due to the high surface-to-volume ratio, many more water molecules can be coordinated to the particle than to molecular Gd-based agents. CS particles have many hydroxyl groups on their surface (see Figure 2) at which bulk water molecules can be temporarily bound and released back to the bulk, contributing to the measured water proton relaxivity r1.

The above-mentioned aspects have been commonly applied by MRI contrast agent researchers to increase r1 relaxivity. For example, many groups have synthesized macrocyclic Gd complexes bound to biomacromolecules or proteins such as BSA, such that the resulting Gd agents have a relatively large rotational correlation time due to their increased size and due to the increased number of coordinated water molecules attached to the Gd complexes on the surface of these biomacromolecules or proteins [42].

## 3. Materials and Methods

### 3.1. Materials

Chondroitin sulfate A sodium salt (CS, ≥98%; average MW, 10,000–30,000; Biosynth carbo synth), iron(III) chloride anhydrous (Fluka, 97%), gadolinium(III) chloride hydrate (Aldrich, 99.99%), zinc(II) chloride anhydrous (purists, Riedel, ≥98%), copper(II) chloride anhydrous (Sigma, ≥98%), dioctyl sulfosuccinate sodium salt (AOT, 96%; Acros Organics), and 2,4-trimethylpentane (isooctane, ≥99.5%; Isolab) were used as received in the synthesis of CS-M(III) and CS-M(II) particles.

In the cell culture study, L929 fibroblast cells (SAP Institute, Ankara, Turkey), Dulbecco’s modified Eagle’s medium (DMEM, L-Glutamine, 15 mM HEPES, 1.2 g/L NaHCO_3_, Pan BioNTech, Aidenbach, Germany), fetal bovine serum (FBS, Pan BioNTech, Aidenbach, Germany), antibiotic (10,000 U/mL penicillin, 10,000 μg/mL streptomycin, Pan BioNTech), and trypsin-EDTA (0.25%, Pan BioNTech) were used as received. Furthermore, trypan blue (0.5% solution, Biological Industries), thiazolyl blue tetrazolium bromide (MTT, BioFroxx), and dimethyl sulfoxide (DMSO, 99.9%, Carlo-Erba) were used for the cytotoxicity analysis. In the antibacterial analysis, *Escherichia coli* ATCC 8739 (KWIK-STIK, Microbiologics) and *Staphylococcus aureus* ATCC 6538 (KWIK-STIK, Microbiologics), and nutrient agar (NA, Condolab, Madrid, Spain) were used as received. All solvents such as acetone and ethanol were of analytical purity. DI water was obtained from a Millipore-Direct Q UV3 (18.2 M·Ω·cm).

### 3.2. Synthesis of CS-Fe(III), CS-Gd(III), CS-Zn(II), and CS-Cu(II) Particles

CS-Gd(III), CS-Fe(III), CS-Zn(II), and CS-Cu(II) particles were synthesized by the physical crosslinking of CS with metal ions according to the procedure described by Sahiner et al. [30]. Briefly, 300 mg of linear CS was dissolved in 10 mL of 0.1 M NaOH aqueous solution and 1 mL of this CS solution was suspended in 30 mL of 0.2 M AOT/isooctane solution at a 1000 rpm mixing rate. After 30 min, 100 μL of metal ion solutions—Gd(III), Fe(III), Zn(II), and Cu(II) solutions prepared in 0.1 M HCl aqueous solution at stoichiometrically the same mol ratio relative to the repeating units of CS—was added into the emulsion medium as a crosslinker. The reaction was stirred at 1000 rpm for 1 h more. After that, the prepared CS-Gd(III), CS-Fe(III), CS-Zn(II), and CS-Cu(II) particles were precipitated in the excess amount of acetone. To remove the unreacted chemicals and surfactant, the precipitated particles were washed with acetone three times via centrifugation at 10,000 rpm for 10 min. The final product was dried in an oven at 50 °C and placed into a closed container for further use.

### 3.3. Characterization of CS-Fe(III), CS-Gd(III), CS-Zn(II), and CS-Cu(II) Particles

The morphological analysis and size distribution of CS-Fe(III), CS-Gd(III), CS-Zn(II), and CS-Cu(II) particles were conducted using a scanning electron microscope (SEM, Quanta 400F field emission SEM) with 10 kV operating voltage after coating with gold to a few nm thicknesses for 30 s. The CS-M microgels were filtered with filter paper with pore size of 5 µm. Then, dynamic light scattering (DLS, Brookhaven Nanobrook Omni, Holtsville, NY, USA) measurements were conducted in 10 mM KNO_3_ to measure the size of the CS-M particles after the filtration. The zeta potential was measured using 40 mg of the CS-M particles suspended in 40 mL of 1 mM KNO_3_ solution using a zeta potential measuring device (Brookhaven Nanobrook Omni, Holtsville, NY, USA). The thermal analysis of linear CS, CS-Fe(III), CS-Gd(III), CS-Zn(II), and CS-Cu(II) particles was evaluated with TGA (SII TG/DTA 6300, Seiko, Tokyo, Japan) at 2 mL/min flow rate of nitrogen gases with 10 °C/min heating rate at 50–750 °C.

### 3.4. Blood Compatibility of CS-Fe(III), CS-Gd(III), CS-Zn(II), and CS-Cu(II) Particles

Blood compatibility of CS-Fe(III), CS-Gd(III), CS-Zn(II), and CS-Cu(II) particles were investigated by hemolysis and blood clotting assays according to ethics committee approval (Human Research Ethics Committee of Canakkale Onsekiz Mart University, 2011-KAEK-27/2022). The fresh blood taken from healthy volunteers was placed into EDTA-containing hemogram tubes.

For the hemolysis test, 1 mL of fresh blood was diluted with 1.25 mL of 0.9% NaCl solution. Then, 0.2 mL of diluted blood was slowly put into a suspension of CS-Fe(III), CS-Gd(III), CS-Zn(II), and CS-Cu(II) particles at a 1 mg/mL concentration in 2 mL of 0.9% NaCl solution. The blood-containing particle suspension was incubated at 37 °C in a shaking water bath. As negative and positive controls, 0.2 mL of diluted blood was placed into 2 mL of 0.9% NaCl solution and DI water, respectively. After 1 h incubation, the suspension was centrifuged at 100× *g* for 5 min and the supernatant was read by UV–Vis spectrophotometer (SP-UV300SRB, Spectrum, Quanzhou, China) at 542 nm. Each particle was analyzed in at least three experiments and the mean value was given with the standard deviation. The hemolysis ratio% was calculated using Equation (4).

Hemolysis ratio% = (A_sample_ − A_negative control_)/(A_positive control_ − A_negative control_) × 100 (4)

For the blood clotting assay, a 100 μL, 10 mg/mL concentration of CS-Fe(III), CS-Gd(III), CS-Zn(II), and CS-Cu(II) particle suspension in 1 mL of 0.9% NaCl solution was placed into flat bottom centrifuge tubes. Then, 0.81 mL of fresh blood was mixed with 0.064 mL of 0.2 M CaCl_2_ solution and 0.27 mL of this solution was slowly dropped onto the particles in the tubes. The tubes were incubated at 37 °C in a shaking water bath. At the end of the 10 min, 10 mL of DI water was slowly added to the samples that contained blood and centrifuged at 100× *g* for 1 min. The supernatant of this solution was gently taken from the tubes and placed on the 40 mL of DI water and incubated at 37 °C in a shaking water bath. As a control group, 0.25 mL of fresh blood was suspended in 50 mL of DI water and incubated under the same conditions. Each particle was analyzed at least three times and the mean value was given with the standard deviation. The absorbance of the sample containing blood and only blood solution as a control was measured using a UV–Vis spectrophotometer at 542 nm. The blood clotting index was calculated according to Equation (5).

Blood clotting index% = (A_sample_/A_control_) × 100 (5)

### 3.5. Cytotoxicity of CS-Fe(III), CS-Gd(III), CS-Zn(II), and CS-Cu(II) Particles

The MTT assay was performed on L929 fibroblast cells to determine the cytotoxicity of the particles. In the culture of the cells, DMEM medium containing 5% FBS and 1% antibiotics was used, and the cells were incubated in a 5% CO_2_/95% air atmosphere at 37 °C. The cells in the growth medium at a 1 × 10^4^ concentration for each well were seeded into a 96-well plate and incubated under the same conditions for 24 h. Then, the medium was removed and 100 μL of the particle suspension into the growth medium at 50–1000 μg/mL concentrations was added into the wells containing attached fibroblast cells. The plate was incubated in a 5% CO_2_/95% air atmosphere at 37 °C for 24 h. Then, the particle solution was removed, and the cells were washed with PBS three times. After that, 100 μL of MTT solution at a 0.5 mg/mL concentration was added to each well and incubated in the dark for 2 h. After decanting the MTT solution, 200 μL of DMSO was added to complete the solubilization of the formazan crystals. The absorbance of the wells was measured at 590 nm to calculate cell viability %. Each particle was analyzed at least three times and the mean value was given with the standard deviation. Statistical analysis was performed using GraphPad Prism 9.0 software by one-way ANOVA followed by non-parametric and Dunn’s multiple comparisons tests. A *p*-value < 0.05 and *p*-value < 0.001 were used to assess significant differences between the experimental and control groups.

### 3.6. Antibacterial Effects of CS-Fe(III), CS-Gd(III), CS-Zn(II), and CS-Cu(II) Particles

Gram-negative *Escherichia coli* ATCC 8739 and Gram-positive *Staphylococcus aureus* ATCC 6538 were used to evaluate the antibacterial activity of the CS-Fe(III), CS-Gd(III), CS-Zn(II), and CS-Cu(II) particles via a microtiter broth dilution assay. The liquid growth medium was nutrient broth (NB), where 100 μL was placed into the wells of a 96-well plate. The particle suspension in the NB from 5 to 0.02 mg/mL concentrations were prepared and 100 μL of this particle suspension was added to the wells. Separately, the bacterial culture at the McFarland standard concentration of 0.5 was prepared and 5 μL of this bacteria stock was added to the wells of a 96-well plate. The plate was incubated at 37 °C for 18–24 h and the minimum inhibition concentration (MIC) was determined as the minimum concentration of antibacterial particle suspension that had no visible growth. Each particle was analyzed at least three times, and the results were presented as the mean average values with the standard deviation.

### 3.7. Magnetic Resonance Imaging (MRI) Analysis of CS-Fe(III), CS-Gd(III), CS-Zn(II), and CS-Cu(II) Particles

In vitro MRI experiments were performed on a phantom including aqueous dispersions of CS-Fe(III), CS-Gd(III), CS-Zn(II), CS-Cu(II), and pristine CS hydrogel microparticles using a NIUMAG Small Animal MRI scanner equipped with 0.5 Tesla permanent magnet (probe size d = 60 mm). The microparticle dispersions were prepared by dissolving each dry gel powder in deionized water at a 0.1 mg/μL concentration. The MR signal was acquired from a 10 mm thick horizontal slice positioned at the center of the sample tubes with 125 mm × 125 mm field-of-view (FOV) and 256 × 256 matrix size using one scan. The T_2_-weighted MR images were recorded using a spin echo (SE) sequence with repetition time TR = 5000 ms and changing echo times TE = 18, 20, 22, 26, 30, 34, 40, 50, 60, 80, 100, 150, 200, 250, and 500 ms in order to obtain the decay curve of the echo signal intensity vs. time. The T_1_-weighted images were recorded using an inversion recovery (IR) sequence with a rephasing pulse following a reading pulse (180°-TI-90°-TE/2-180°-TR) with the repetition time TR = 3000 ms and echo time TE = 20 ms, and in order to obtain the signal time evolution of the signal (i.e., the saturation curve), the inversion time was changed to TI = 2, 4, 6, 8, 10, 15, 20, 50, 100, 150, 500, 750, 1000, 1250, 1500, and 2000 ms. Both sets of T_1_-weighted and T_2_-weighted images were analyzed using the RadiAnt DICOM Viewer program.

## 4. Conclusions

Here, anionic CS as a natural polymer in a basic condition was directly crosslinked by trivalent Fe(III) and Gd(III) cations or divalent Zn(II) and Cu(II) cations under acidic conditions. The metal ion solution of 0.1 M was used to prevent precipitation in hydroxide forms, which were neutralized when mixed with a 0.1 M NaOH solution of CS. The prepared CS-Fe(III), CS-Gd(III), CS-Zn(II), and CS-Cu(II) particles were spherical in shape with a size range of micrometers to a few hundred nanometers because of the microemulsion technique used to generate these polymeric particles. These CS–metal ion particles were assessed as being hemocompatible according to the hemolysis and blood clotting tests, implying their safe use in intravenous applications. Moreover, the CS-Fe(III), CS-Gd(III), and CS-Cu(II) particles revealed less toxicity on fibroblasts even at a concentration of 1000 μg/mL. The antibacterial study results indicated that CS-Zn(II) and CS-Cu(II) particles are particularly potent and very effective in eradicating Gram-negative and Gram-positive bacteria species and could be utilized as antibacterial additive biomaterials. The MRI studies revealed that amongst the CS–metal ion particles as a measure of MRI contrast enhancement efficiency, CS-Gd(III) shows the highest r_1,2_ relaxivity values, even higher than commercial Gd-based contrast agents. Nevertheless, except non-magnetic CS-Zn(II), all particles have the potential to increase the image contrast in MR imaging and they act as negative contrast agents with r_2_/r_1_ > 2, much like superparamagnetic nanoparticles.

So, the CS-based microgels prepared here are versatile materials that have encouraging potential for in vivo applications as blood-compatible, antibacterial, biocompatible, and MRI contrast-enhancing agents.

## Figures and Tables

**Table 1:** Antibacterial activity of CS-Fe(III), CS-Gd(III), CS-Zn(II), and CS-Cu(II) particles by minimum inhibition concentration (MIC) values against Gram-negative *Escherichia coli* ATCC 8739 and Gram-positive *Staphylococcus aureus* ATCC 6538 for a 24 h incubation time.

Particles	Minimum Inhibition Concentration (MIC, mg/mL)	
	*E. coli*	*S. aureus*
CS-Fe(III)	5.0	N.D. ^1^
CS-Gd(III)	N.D.^1^	N.D. ^1^
CS-Zn(II)	2.5	5.0
CS-Cu(II)	5.0	5.0

^1^ N.D.—not determined.

**Table 2:** List of T_1_,_2_ values for CS–metal and CS microgel suspensions obtained from mono exponential fits in Figure 7 and proton relaxivities r_1,2_ calculated according to Equation (3). (C: metal ion concentration).

	C (mM)	T_1_ (ms)	T_2_ (ms)	r_1_ (s^−1^·mM^−1^)	r_2_ (s^−1^·mM^−1^)
CS-Gd(III)	0.96	77.3 ± 4.9	8.7 ± 0.6	13.1	117.4
CS-Fe(III)	3.12	537.3 ± 19.3	24.7 ± 3.1	0.49	12.3
CS-Cu(II)	1.56	357.0 ± 21.3	83.2 ± 5.2	1.58	6.29
CS-Zn(II)	1.94	1763.5 ± 56.3	245.6 ± 41.7	0.12	0.96
CS	-	3083.0 ± 519.4	453.8 ± 173.2	-	-

## Data Availability

Data is contained within the article and supplementary material.

## Supplementary Materials

The following supporting information can be downloaded at: https://www.mdpi.com/article/10.3390/ph16040483/s1. Figure S1: An example of selected ROIs in order to read the signal intensities on MRI image in Ra-diAnt DICOM Viewer (Image obtained with SE sequence TR = 5000 ms TE = 30 ms).
