# Supplementary material for: Physically Crosslinked Chondroitin Sulfate (CS)–Metal Ion (M: Fe(III), Gd(III), Zn(II), and Cu(II)) Particles for Versatile Applications and Their Biosafety
Source: Pharmaceuticals (Basel). 2023 Mar 23;16(4):483. doi: 10.3390/ph16040483 (PMC10144968; doi:10.3390/ph16040483)
Supplement: Supplementary file 1 [file pharmaceuticals-16-00483-s001.zip › pharmaceuticals-2172639-supplementary.pdf]

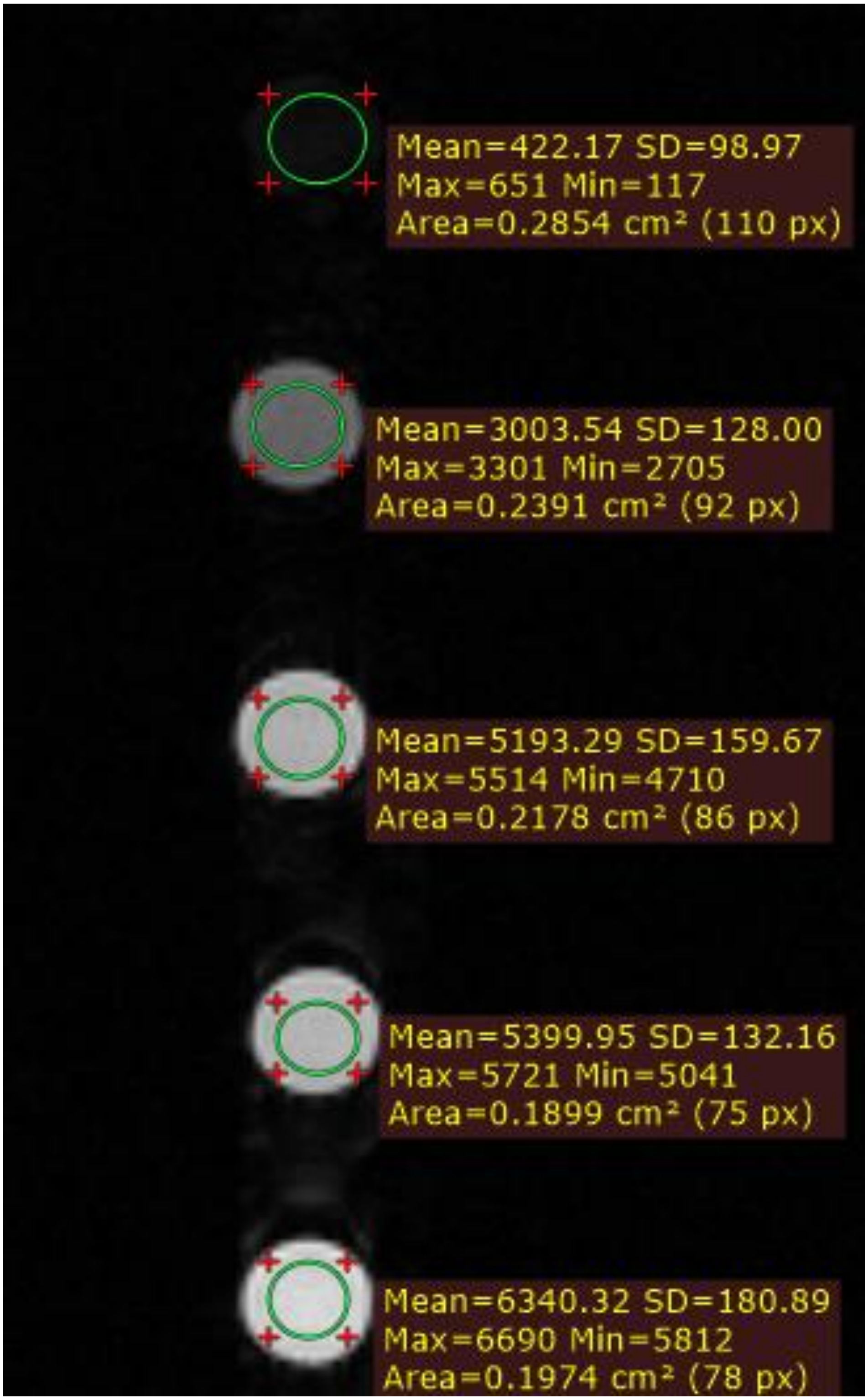

Figure S1. An example of selected ROIs in order to read the signal intensities on MRI image in RadiAnt DICOM Viewer (Image obtained with SE sequence TR=5000 ms TE=30 ms).
